# Decreasing incidence of cutaneous chemical burns in a resource limited burn centre: is this a positive effect of modernization?

**DOI:** 10.1186/s41038-017-0072-1

**Published:** 2017-03-07

**Authors:** R. E. E. Nnabuko, C. P. Okoye, I. S. Ogbonnaya, Egi Isiwele

**Affiliations:** 1 Burns and Plastic Department, National Orthopaedic Hospital, PMB 01294 Enugu, Nigeria; 2 Department of Surgery, University of Calabar Teaching Hospital, Calabar, Nigeria

**Keywords:** Chemical burns, Interpersonal assault, Lead-acid battery vendors, Resource-limited country, Modernization

## Abstract

**Background:**

Burns present a devastating injury to patients. Burns caused by chemical agents, present a worse scenario. In a resource limited country like Nigeria, readily available sources of these corrosive agents are mainly from lead-acid battery vendors and to some extent local small scale soap manufacturers who use caustic soda. We hypothesized that with the reduction in small scale soap manufacturing and increasing trend towards modernization in the use of dry cell batteries, chemical burns may be on the decline, and we sought to investigate this.

**Methods:**

The records of all acute burn patients seen at the Burns and Plastic Department of the National Orthopaedic Hospital Enugu Nigeria between January 2011 and December 2014 were retrospectively reviewed. The results were compared to similar studies carried out at the same centre. A questionnaire was administered to corrosive chemical (sulphuric and caustic soda) vendors to assess the trends in product sales and use in recent times.

**Results:**

A total of 624 acute burn cases were treated during the period; among which, 12 cases (1.9%) were chemical burns. When compared with previous studies at the centre, Chemical burn cases were  recorded as the lowest rate. The median age of patients was 24 years. There were eight males and four females. Interpersonal assault was the commonest mechanism of injury with sulphuric acid suspected to be the commonest agent in 83.3% of the cases, while 16.7% of the cases were from accidental use of caustic soda. The head and neck as well as the upper limbs were the most affected (30%).

Twenty-six questionnaires to lead-acid vendors were analyzed and revealed that all respondents noticed a marked downward trend in the sale of either sulphuric acid or caustic soda, and they attributed this to the ready availability of imported alternatives to locally manufactured soap or wet lead-acid batteries. Ease of use, durability and convenience of the dry cell batteries were cited as principal reasons.

**Conclusion:**

There appears to be a downward trend in the prevalence of chemical burns in our study compared to previous studies in the centre which may be due to reduced availability and access of corrosive chemicals to the general public. Further prospective multicentre studies to confirm this are recommended.

**Electronic supplementary material:**

The online version of this article (doi:10.1186/s41038-017-0072-1) contains supplementary material, which is available to authorized users.

## Background

Cutaneous burns are among the most devastating injuries to the human body [1]. This is not unconnected to the imprecise nature of this injury and the tremendous attendant tissue destruction that lies in its wake.

Cutaneous chemical burns complicate this picture producing the worse disfigurement leading to various forms of post-burn deformities. Chemical burns can arise from industrial accidents, explosions and assaults amongst other mechanisms. In low and middle income countries of Africa and South East Asia with little or moderate industrialization, most of the cases of chemical injuries are related to assault [2, 3]. The chemicals implicated include acids and alkalis amongst other substances with the degree of injury dependent on concentration, duration of contact, and nature of the agent [4].

In Nigeria, the common sources of these chemicals, following interaction with persons accused of using such, suggests that wet lead-acid battery vendors provide readily available source for acids while traditional soap makers are the major outlet for caustic alkalis. The demand for acids was further boosted in the past by certain local sanitary practices where sulphuric acid was the popular agent used to oxidize waste in pit latrines and curing tapped rubber. These economic and social behaviors of modernization might have accounted for the apparent increase in the ready availability and incidence of chemical burns according to some studies [5, 6].

In the recent past however, we have observed a decline in the presentation of chemical burn injury to our burn service which is a regional burn centre in South East Nigeria. This led us to theorize that improvements in life style, adoption of western lifestyles and the gradual demise of local soap manufacturing industries due to more fashionable and economically viable options might have reduced access of the general populations to these dangerous chemicals. The increasing preference and availability of dry cell batteries might also have played a role.

We therefore decided to test our hypothesis by retrospectively analyzing chemical burns presentation to the hospital in the recent past. To further test our hypothesis, questionnaires were produced and self-administered to vendors of corrosive chemicals to determine the current trend in the sale of these products.

## Methods

The study was conducted in two parts. In the first part, records of all the patients presenting with cutaneous burns to the hospital were extracted and reviewed retrospectively from case notes, the emergency room, and theatre records at the National Orthopaedic Hospital Enugu, South East Nigeria between January 2011 and December 2014. The records of patients with chemical burns were further analyzed, and data relating to age, sex, mechanism of injury, type of chemical, total burn surface involved and region of the body involved were extracted. Results obtained were compared with other studies from the same centre.

In the second part, a questionnaire was designed and self-administered prospectively to vendors of lead-acid batteries and these chemicals (Additional file 1). Participants were selected by convenience sampling because their business are located in a specific location in town. These questionnaires assessed formal training in handling chemicals among vendors, storage of these chemicals, knowledge of safety procedures for vendors and customers and the trend in corrosive chemicals sales dynamics over the period under review.

## Results

A total of 624 patients were treated for cutaneous burns from different aetiologies (Table 1) during the period under review. Chemical injuries accounted for 1.9% (*n* = 12) of all the cases. The age range of the patients with chemical burns was from 20 to 65 years and the median age was 24 years.

**Table 1:** The distribution of burn aetiology within the period

Burn aetiology	Number of patients, n (%)
Scald	181 (29.0%)
Friction	42 (6.7%)
Flame	367 (58.9%)
Electrical	22 (3.5%)
Chemical	12 (1.9%)
Total	624 (100.0%)

Among these subjects with chemical injuries, there were eight male and four female patients. In 10 (83.3%) of these cases, the type of chemical agent involved was suspected to be sulphuric acid (Table 2). Interpersonal assault was the major cause of the injuries accounting for 83.3% (*n* = 10) of the injuries sustained. Accidental burns were encountered in the use of caustic alkalis in two (16.7%) cases.

**Table 2:** The agents implicated and the mechanism of injury

Agent	Frequency	Mechanism of injury	Percentage (%)
Sulphuric acid	10	Interpersonal assault	83.3
Caustic soda	2	Accident	16.7
Total	12		100.0

As regards the region of the body involvement, it was observed that the head and neck regions of the body and the upper limb were the most affected areas (Table 3).

**Table 3:** Sites affected by chemical burn injury

Area of body involved	Number of patients, n (%)
Head and neck	9 (30.0%)
Trunk	7 (23.3%)
Upper limbs	9 (30.0 %)
Lower limbs	5 (16.7%)
Perineum	0

Twenty-six (26) questionnaires were distributed and administered by the authors to vendors of sulphuric acid and caustic soda. These agents were used by lead-acid battery chargers and small scale industry soap manufacturers respectively. Twenty one (80.8%) respondents marketed sulphuric acid and five (19.2%) marketed caustic soda. The demographic analyses revealed that the respondents were all males with an average age at 35.7 years. Sixteen respondents (61.5%) had secondary school education while 10 (38.5%) had pre-secondary school education. The mean duration of involvement in the trade was 9.6 years.

There was generally no formal training in the handling of these chemicals which in almost all cases were not stored in special containers but were stored in large plastic jars and drums without any safety measures. Accidents therefore are liable to occur. Vendors did not regulate who the products were sold to as anyone was free to make a purchase. Sulphuric acid was sold mainly to lead-acid battery chargers while caustic soda was sold to small scale manufacturers of soap.

All the respondents had noticed a marked downward trend in the sale of corrosive chemicals in the period under review. The principal reasons advanced for this trend were that consumers had other readily available and cheaper alternatives making these chemicals less available. Availability was also negatively affected by increasing importation tariffs and more regulating authority control (Table 4). These vendors also explained that dry cell batteries were easier and more convenient to use and generally cheaper than wet lead-acid batteries.

**Table 4:** Reasons for downturn in sales of chemicals

Reasons for downturn in sales of chemicals	Decreasing order of importance (scale of 1–5)
Consumers have other alternatives	5
Reduced availability	4
Rising costs	3
Increasing importation tariffs	2
Increase in regulating authority control	1

## Discussion

The frequency of accidental acid burns in developed societies of North America and Europe are considered low [6–10]. In some developing countries, chemical burns are related to assault and are fairly quite common. Literature has revealed acid burn incidence of 17% in Uganda [2] and 20% in Cambodia [11].

In Nigeria, studies have suggested that chemical burns particularly from interpersonal assault are common. In the 1980s, Achebe and Akpuaka [12] documented a prevalence rate of 3.3%. Olaitan et. al [5] in 2008 documented that over a 4-year period (2000–2003) chemical injuries from assault accounted for 5.7% of cases. Nnabuko et. al [13]. in Enugu, established that chemical burns made up 6.3% of all patients presenting with burn between 2000 and 2005. These three quoted studies were done at the Burns Centre of the National Orthopaedic Hospital, Enugu, Nigeria (Table 5). Other studies outside Enugu have also suggested that chemical burns were common presentation [14].

**Table 5:** Comparison of cutaneous chemical burns from different studies in the centre

Study	Study duration	Total number of cutaneous burns	Total number of chemical burns, n (%)
Achebe and Akpuaka [12]	Jan.1981–Dec. 1983	239	8 (3.3%)
Olaitan and Jiburum [5]	Jan. 2000–Dec. 2003	485	28 (5.8%)
Nnabuko et al [13]	Jan. 2002–Dec. 2005	414	26 (6.3%)
Nnabuko et al (index study)	Jan. 2011–Dec. 2014	624	12 (1.9%)

However, in the course of our practice lately, it was observed that the number of patients presenting to our accident and emergency department with chemical injuries appeared to have been on the decline. Since the hospital is a regional burn centre, we sought to investigate this observation. Our study revealed that over a 4-year period, chemical injuries accounted for 1.9% at all the burn cases. This finding is at variance with other studies from the same centre which had documented much higher values [5, 12, 13].

These findings we hypothesized may not be unconnected with changing lifestyles, technological transfer and the apparent demise of the local industries. It was noted that most of the cases from the series were due to interpersonal assault. The sources of these agents for these nefarious activities, we deduced from interviews of members of the public, were from lead-acid battery vendors as well as those involved in traditional soap making with palm oil and caustic soda. The traditional soap-making industry has witnessed a decline due to decreasing palm production [15] as well as availability of cheaper alternatives. Social practices where acids were used to hasten the decomposition in pit latrines are also on the decline due to the improving sanitary conditions brought about by abolishment of pit latrines in urban areas and many rural communities. These practices reduced the need to procure some of these chemical. Furthermore, the use of battery acid electrolyte, a by-product of dilution of sulphuric acid, has sharply declined as a result of increased use of dry cell batteries.

Our study revealed a marked decline in the sale of corrosive chemicals. Sulphuric acid and caustic soda are the commonest corrosive agents in common use, and most chemical burns in our environment are caused by these two agents. The introduction of cheaper and more effective alternatives and the rising costs of these agents have caused reduced access to these agents to people who may wish to use them for criminal activities. We therefore believe that improved lifestyles and improved technology has a positive bearing on the reduced accessibility to corrosive chemicals.

It is pertinent to point out that this observation was based on the database of only one burn centre in the country and only over a 4-year period. These factors would be a limitation to the study. For a more reliable conclusion, this study should be carried out in more burn centres in the local environment and over a longer study period.

## Conclusions

There does appear to be a downward trend in the prevalence of chemical injuries in our study compared with previous studies from the centre. This may be attributed to the improving technological and economic developments in the country with the subsequent change in lifestyle. Other combinations of regulating activities on handling and sale of chemicals that make them relatively inaccessible to the criminal minded may have been involved. We believe however that these factors applied on a large scale will also see the decline of the incidence of burn injuries as a whole in low and middle income countries.

## Additional file

Additional file 1: Questionnaire. (DOCX 55 kb)

## References

[1] Sen SL, Rashid MA (2003). Burn cases: a medical and social problem in Bangladesh. Orion Med J.

[2] Asaria J, Kobusingye OC, Khingi BA, Balikuddembe R, Gomez M, Beveridge M (2004). Acid burns from personal assault in Uganda. Burns.

[3] Ramakrishna K, Mathivanan J, Jayaraman V, Babu M, Shankar J (2012). Current scenario in chemical burns in a developing country: Chennai, India. Ann Burns Fire Disasters.

[4] Palao R, Monge I, Ruiz M, Barret JP (2009). Chemical burns: pathophysiology and treatment. Burns.

[5] Olaitan PB, Jiburum BC (2008). Chemical injuries from assault: an increasing trend in a developing country. Indian J Plast Surg.

[6] Leonard LG, Scheulen JY, Munster AM (1982). Chemical burns effects at prompt first aid. J Trauma.

[7] Sykes RA, Moni MM, Hiebert JM (1986). Chemical burns: retrospective review. J Burn Care Rehabil.

[8] Mozingo DW, Smith AA, Mc Manus WF, Pruitt BA, Manson AD (1988). Chemical burns retrospective review. J Trauma.

[9] Trenel H, Brunier A, Walmann LS (1991). Chemical burns caused by hydrofluoric acid: incidence, frequency and current state of therapy. Med Klin.

[10] Munnoch DA, Darcy CM, Whallet EJ, Dickson WA (2000). Work-related burns in South Wales 1995–1996. Burns.

[11] Ly H, Sarom N, Gollogly J, Beveridge M (2001). 88 burns operated at ROSE rehabilitation centre, Phnom Penh, paper read at 7^th^ Annual Cambodian Surgical Congress.

[12] Achebe UJ, Akpuaka FC (1989). Chemical burns in Enugu. West Afr J Med.

[13] Nnabuko REE, Ogbonnaya IS, Otene CI, Ogbonna U, Amanari OC, Opara KO (2008). Burn injuries in Enugu, Nigeria –aetiology and prevention. A Six year retrospective review. Ann Burns Fire Disaster.

[14] Dongo AE, Irekpita EE, Oseghale LO, Ogbebor EE, Iyamu EE, Onuminya JE, et al. A fire year review of burn injuies in Irrua. BMC Health Serv Res. 2007;171–177. 10.1186/1472-6963-7-171 PMC2174937 17956614

[15] Ibitoye OO, Akinsorotan AO, Meludu NT, Ibitoye BO (2011). Factors affecting oil palm production in Ondo state of Nigeria. J Agric Soc Res.

