# Supplementary material for: Decreasing incidence of cutaneous chemical burns in a resource limited burn centre: is this a positive effect of modernization?
Source: Burns Trauma. 2017 Mar 7;5:7. doi: 10.1186/s41038-017-0072-1 (PMC5341456; doi:10.1186/s41038-017-0072-1)
Supplement: Additional file 1: — Questionnaire. (DOCX 55 kb) [file 41038_2017_72_MOESM1_ESM.docx]

**BURNS AND PLASTIC DEPARTMENT NATIONAL ORTHOPAEDIC HOSPITAL ENUGU**

**QUESTIONNAIRE**

TITLE-**Attitude and practice of vendors in Enugu, Nigeria towards use and sale of corrosive chemicals**

This survey is for academic purposes and no personal data will be recorded.

**BIODATA**

A. Age(in years):

B. Sex: male female

C. Educational level: Pre Secondary Secondary School Post Secondary

**PRACTICE AND ATTITUDE**

1. What type of chemicals do you trade on**? acid caustic soda**

2. Number of years in chemicals business:

3. Did you have any formal training on the handling of the products you sell?

Yes No

4. How do you store the products: plastic cans special containers

5. In the past 3 years, what has the volume of sales been like : A. Increasing

B. static C. Diminishing

6. What are the possible reason(s) affecting your volume of sales. Indicate in order of importance.

A. cost B. Reduced availability C. Increasing importation tariffs

D. Increasing control by regulating authorities E. Consumers have other

alternatives

7. Briefly explain your response in ‘5” above ………………………………………………………………………………………………………………………………………………………………………………………………………………………………………………………………………………………………………………………………………………………………………………………………………………………………………………………………………………………

8. Do you self-regulate whom you sell your products to: yes no

9. If you self-regulate, what class of customers do you sell to (you can choose more than one option)

A. Rubber tappers

B. Chemists

C. Pit latrine cleaners

D. Lead acid battery chargers

E. Small scale soap makers

F. Others (specify)

10. Do you think there is need for government regulation on chemicals sale: yes no

11. Are you aware of injuries these chemicals cause when used on humans: yes no

12. Have you ever sustained such injuries in the course of your business? Yes no

12. If your answer is yes, do you advice your customers on safety precautions? Yes no

Thank you.
